# Conscientiousness is modified by genetic variation in catechol-*O*-methyltransferase to reduce symptom complaints in IBS patients

**DOI:** 10.1002/brb3.294

**Published:** 2014-11-13

**Authors:** Kathryn T Hall, Benjamin R Tolkin, Garrett M Chinn, Irving Kirsch, John M Kelley, Anthony J Lembo, Ted J Kaptchuk, Efi Kokkotou, Roger B Davis, Lisa A Conboy

**Affiliations:** 1 Program in Placebo Studies, Beth Israel Deaconess Medical Center and Harvard Medical SchoolBoston, Massachusetts; 2 Division of General Medicine and Primary Care, Beth Israel Deaconess Medical Center and Harvard Medical SchoolBoston, Massachusetts; 3 Department of Medicine, Massachusetts General Hospital and Harvard Medical SchoolBoston, Massachusetts; 4 School of Psychology, University of PlymouthDevon, UK; 5 Department of Psychiatry, Massachusetts General Hospital, Harvard Medical SchoolBoston, Massachusetts; 6 Endicott CollegeBeverly, Massachusetts; 7 Department of Gastroenterology, Beth Israel Deaconess Medical Center and Harvard Medical SchoolBoston, Massachusetts

**Keywords:** Catechol-*O*-methyltransferase, complaints, COMT, conscientiousness, personality

## Abstract

**Background:**

Attention to and perception of physical sensations and somatic states can significantly influence reporting of complaints and symptoms in the context of clinical care and randomized trials. Although anxiety and high neuroticism are known to increase the frequency and severity of complaints, it is not known if other personality dimensions or genes associated with cognitive function or sympathetic tone can influence complaints. Genetic variation in catechol-*O*-methyltransferase (COMT) is associated with anxiety, personality, pain, and response to placebo treatment. We hypothesized that the association of complaint reporting with personality might be modified by variation in the COMT val158met genotype.

**Methods:**

We administered a standard 25-item complaint survey weekly over 3-weeks to a convenience sample of 187 irritable bowel syndrome patients enrolled in a placebo intervention trial and conducted a repeated measures analysis.

**Results:**

We found that complaint severity rating, our primary outcome, was negatively associated with the personality measures of conscientiousness (β = −0.31 SE 0.11, *P *=* *0.003) and agreeableness (β = −0.38 SE 0.12, *P *=* *0.002) and was positively associated with neuroticism (β = 0.24 SE 0.09, *P *=* *0.005) and anxiety (β = 0.48 SE 0.09, *P *<* *0.0001). We also found a significant interaction effect of COMT met alleles (β = −32.5 SE 14.1, *P *=* *0.021). in patients genotyped for COMT val158met (*N*  = 87) specifically COMT × conscientiousness (β = 0.73 SE 0.26, *P *=* *0.0042) and COMT × anxiety (β = −0.42 SE 0.16, *P *=* *0.0078) interaction effects.

**Conclusion:**

These findings potentially broaden our understanding of the factors underlying clinical complaints to include the personality dimension of conscientiousness and its modification by COMT.

## Introduction

Complaints in clinical practice are unwanted physical, mental, or emotional symptoms (Wells and Kaptchuk 2012) associated with anxiety and neuroticism (Costa and McCrae 1987). In randomized clinical trials (RCTs), these same complaints can lead to nonadherence, treatment failure, and treatment discontinuation by physicians and/or patients (Mitsikostas et al. 2011). Genetic variation in catechol-*O*-methyltransferase (COMT) has been shown to be associated with anxiety, personality (Stein et al. 2005), pain (Diatchenko et al., 2006) and response to placebo treatment (Hall et al. 2012, Wendt, et al., 2014). We hypothesized that the association of complaint reporting with personality might be modified by variation in the COMT val158met genotype.

The presence of complaints in the placebo arm of trials suggests that in some cases symptoms are not solely attributable to drug treatment, and are often independent of healthcare provider treatment and RCT enrollment (Reidenberg and Lowenthal 1968). In RCTs, factors that contribute to the reporting of these complaints include increased awareness of and attention to preexisting conditions, somatization of expected drug side effects which are often associated with disclosures during informed consent (Wells and Kaptchuk 2012) and the elicitation of symptoms as part of the data collection process, worsening of disease, mild exacerbation of subjective symptoms (e.g., fatigue), emotional symptoms (e.g., anxiety) (Barsky et al. 2002; Rief et al. 2006; Amanzio et al. 2009), and psychological characteristics such as anxiety and the personality dimension of neuroticism (Costa and McCrae 1987). The tendency to somaticize has been associated with temperament in some studies (Hyphantis et al. 2013) but not in others (Karvonen et al. 2006), and symptom reporting is in turn influenced by affective states even in the absence of a difference in symptom severity (Skotzko 2009). Although the total system that gives rise to symptom complaints is not fully understood, personality may play a broad role, either by altering symptom severity, symptom perception, or desire to report.

This network of affective, personality, and cognitive states is rendered more complex by potential genetic variation in genes such as COMT which is involved in mediating sympathetic and dopaminergic tone through the catecholamine pathway. COMT degrades catecholamines such as dopamine, epinephrine, and norepinephrine, and has been shown to influence anxiety and personality. Genetic variation at the COMT val158met locus has been extensively studied because this functional polymorphism results in a three- to fourfold reduction in the activity of the methionine (met) form of the enzyme as compared to the valine (val) form and this activity is inversely correlated with catecholamine levels. COMT val158met genetic variation has also been associated with sensitivity to pain (Diatchenko et al. 2006), cognitive and executive functions (Meyer-Lindenberg et al. 2005), and response to placebo treatment (Hall et al. 2012). Associations between COMT genotype and personality traits have also been reported in several studies (Stein et al. 2005; Aoki et al. 2011).

Using a convenience sample from a previously reported irritable bowel syndrome (IBS) clinical trial (Kaptchuk et al. 2008), we investigated psychological and genetic correlates of complaint reporting independent of treatment. IBS is a chronic condition characterized by abdominal pain, bloating, and altered bowel function (Somers and Lembo 2003). Although few patients in this trial attributed their complaints to the study (Kaptchuk et al. 2008), placebo response was associated with COMT val158met (Hall et al. 2012). Here, we examine the relationship between anxiety and the personality traits neuroticism, conscientiousness, agreeableness, extraversion, and openness (McCrae and Costa 1987) on complaint ratings and the interaction of these personality dimensions with variation at COMT val158met.

## Materials and Methods

### Subjects and clinical trial design

Details of the design and outcomes of this IBS placebo trial are provided elsewhere (Conboy et al. 2006; Kaptchuk et al. 2008). Briefly, the parent RCT investigated the effects of placebo acupuncture on IBS symptoms. No active medication was given. Enrolled patients were ≥18 years old and diagnosed by Rome II criteria for IBS with a score of >150 on the IBS Symptom Severity Scale (IBS-SSS), *n* = 262 (Francis et al. 1997). Participants were recruited from advertisements and flyers and through referrals from health professionals. A board-certified gastroenterologist confirmed the diagnosis of IBS. Patients were excluded if they had weight loss greater than 10% of body weight, fever, blood in stools, a family history of colon cancer, inflammatory bowel disease, significant psychiatric comorbidities, or prior acupuncture treatment. Patients were allowed to continue taking medications for IBS if the therapeutic regimen had remained constant for the previous 30 days, and they agreed to keep the regimen constant during the trial. A subset of the patients enrolled in the trial (*N* = 187) completed at least one complaint survey over the 3-week trial period. The Institutional Review Board at Beth Israel Deaconess Medical Center (Boston, MA) approved this study which was conducted in accordance with the Declaration of Helsinki.

### Self-report measures

A survey of 25 common complaints (Reidenberg and Lowenthal 1968) was administered weekly to IBS patients enrolled in the IBS placebo trial. Patients were asked to rate the severity of their complaints on a scale of 1 to 5 (5 being the most *severe*) and to indicate whether they attributed the complaint to the study. The primary outcome in this study, complaint severity rating, was the sum of all the severity ratings and ranged theoretically from 0 for no side effects to 125 for all side effects with the maximum severity. Since this standardized index included symptoms typically associated with IBS, such as constipation and diarrhea, the questionnaire was modified to ask whether the patient experienced “more constipation” or “more diarrhea.” Complaints attributed to the placebo (sham acupuncture) intervention (i.e., side effects perceived to be due to treatment) were reported elsewhere (Kaptchuk et al. 2008). Neuroticism, conscientiousness, agreeableness, openness to experience, and extraversion were assessed using the NEO Five-Factor Inventory (NEO-FFI). Anxiety was measured using the Beck Anxiety Scale (Beck et al. 1988), and somatization was assessed using the Whitely index (Pilowsky 1967).

### Genotyping

Genomic DNA was extracted from whole blood samples from patients who gave consent for genetic analysis (*N* = 112) using the Qiagen Blood Kit (Valencia, CA) following the manufacturer's protocol. Eighty-seven patients who reported complaints also consented to be genotyped. TaqMan SNP Genotyping assay for COMT val158met (rs4680) was purchased from Applied Biosystems (Foster City, CA). Quantitative PCR was performed at the Biopolymers Facility at Harvard Medical School (Boston, MA), following the manufacturer's protocol on an Applied Biosystems 7900HT instrument, using SDS version 2.4 software.

### Statistical analysis

To evaluate associations between personality, baseline anxiety, somatization (Whitely Index), IBS-SSS, age, and gender with the primary outcome complaint severity rating across the 3 weeks of the trial we used generalized estimating equations (GEE) to carry out a repeated measures general linear model procedure analysis. Within-subject correlation structure was set as exchangeable and week number was included as a covariate. A subanalysis of patients genotyped at COMT val158met was carried out to examine the interaction between significant personality measures and COMT genotype, using the same statistical parameters for personality and dividing patients into met allele carriers or val/val homozygotes. Hardy–Weinberg equilibrium (HWE) for genotype frequency was calculated using the Online Encyclopedia for Genetic Epidemiology studies (http://www.oege.org/software/hwe-mr-calc.shtml) (Rodriguez et al. 2009). Data were analyzed using SAS Version 9.3 (SAS Institute Inc., Cary, NC).

## Results

Most of the patients in the trial who completed the weekly complaint survey (*N* = 187) were women (78%), with an average age of approximately 39 ± 15 years. Less than half were married or living with a partner (43%), and the majority were white (89% of the total sample, and 94% of those genotyped) (Table 1). The comparatively higher percentage of white subjects carrying met alleles is consistent with current estimates of global COMT allele frequencies (Palmatier et al. 1999). Baseline IBS symptom severity scale and psychological measures are given in Table 1.

**Table 1:** Demographics and baseline characteristics

		COMT genotyped subset (*N* = 82)		
	All patients (*N* = 187)	met/met (*n* = 17)	val/met (*n* = 42)	val/val (*n* = 23)
Demographics				
Age in years, mean	39 (15)	39 (13)	37 (13)	40 (14)
Women (%)	145 (78)	14 (82)	33 (79)	18 (79)
White (%)	164 (89)	17 (100)	39 (93)	21 (91)
Married/living together (%)	69 (43)	8 (53)	15 (47)	8 (38)
IBS severity and psychological measures				
Baseline IBS-SSS	278 (72)	298 (83)	263 (69)	270 (61)
Beck anxiety	12.3 (8.9)	14.3 (7.6)	13.0 (10.9)	12.3 (9.1)
Whitely somatization	2.2 (2.0)	2.6 (1.6)	1.9 (2.1)	2.5 (1.9)
Conscientiousness^1^	47.0 (13.6)	44.3 (13.8)	47.8 (13.3)	47.3 (13.1)
Neuroticism	52.2 (11.7)	55.7 (12.1)	51.9 (12.2)	52.2 (10.9)
Agreeableness	51.7 (12.5)	50.6 (11.8)	51.2 (13.2)	54.2 (11.0)
Extraversion	53.1 (12.1)	49.2 (13.1)	53.6 (12.5)	53.0 (10.9)
Openness	57.9 (11.4)	55.5 (11.4)	59.4 (11.1)	56.1 (10.5)

All numbers are *n* (SD) unless otherwise indicated.

1 Five factor inventory scores are reported as *T*-scores.

Although the number of respondents varied across the 3 weeks (week 1, *N* = 152; week 2, *N* = 185; and week 3, *N* = 126), the responses were consistent and highly correlated (Table 2). There was no significant difference in complaint severity rating between genotyped subjects and subjects who were not genotyped (*P *=* *0.82). Very few patients categorized their complaints as a study-related side effect. The complaint most frequently attributed to the trial placebo interventions was increased constipation (4%). The most frequent complaint was fatigue, with over 50% of patients reporting fatigue across all 3 weeks (Table 2). Other commonly reported complaints were headache, irritability, inability to concentrate, and nasal congestion. Vomiting and excessive bleeding were the least frequently reported complaints across all 3 weeks.

**Table 2:** Mean proportion of respondents in weeks 1 (*N* = 152), 2 (*N* = 185), and 3 (*N* = 126) who reported each of the 25-items on the Complain Severity Scale

Side effect	Mean among weeks	SD
Fatigue	0.60	0.01
Headache	0.48	0.03
Irritability	0.48	0.03
Nasal congestion	0.44	0.06
Inability to concentrate	0.40	0.04
Excessive sleepiness	0.33	0.02
More constipation	0.32	0.05
Insomnia	0.32	0.01
Dry mouth	0.30	0.02
Bad dreams	0.29	0.02
Faint or dizzy when stand	0.29	0.03
Pain in muscles	0.28	0.02
More diarrhea	0.28	0.02
Nausea	0.28	0.03
Pain in joints	0.27	0.02
Loss of appetite	0.20	0.01
Weakness	0.18	0.04
Bruising	0.15	0.02
Palpitations	0.13	0.02
Bleeding from gums after brushing	0.13	0.02
Skin rash	0.12	0.01
Giddiness	0.10	0.03
Fever	0.09	0.02
Vomiting	0.04	0.02
Endorse excessive bleeding	0.02	0.01

In an unadjusted repeated measures model we found that our primary outcome, complaint severity rating, was positively associated with somatization as measured by the Whitely Index of hypochondriasis (*β *= 1.81, SE 0.40, *P *<* *0.0001), anxiety as measured by the Beck Anxiety Scale (*β *=0.48, SE 0.09, *P *<* *0.0001), and the patient's baseline IBS symptom severity scale (β = 0.03, SE 0.01, *P *=* *0.001) (Table 3). Complaint severity rating was positively associated with the NEO Five-Factor Inventory (NEO-FFI) personality dimensions of neuroticism (β = 0.24, SE 0.09, *P *=* *0.005). In addition we found that complaint severity rating was negatively associated with conscientiousness (β = −0.31, SE 0.11, *P *=* *0.003) and agreeableness (β = −0.38, SE 0.12, *P *=* *0.002) but not openness to experience (β = −0.08, SE 0.13, *P *=* *0.5) or extraversion (β = −0.16, SE 0.13, *P *=* *0.2) (Table 3). Since conscientiousness and agreeableness were also associated with complaint severity rating we further examined the correlation of these personality dimensions with neuroticism. We found that conscientiousness (*r* = −0.41, *P *<* *0.0001) and agreeableness (*r* = −0.39, *P *<* *0.0001) were both inversely correlated with neuroticism; conscientiousness and agreeableness were also correlated with each other (*r* = 0.30, *P *<* *0.0001). Complaint severity rating was not associated with age or gender (Table 3).

**Table 3:** Unadjusted repeated measures analysis of main effects on primary outcome sum complaint severity rating

Covariate	β (SE)	*P*-value
Personality (big five)		
Conscientiousness	−0.31 (0.11)	0.003
Neuroticism	0.24 (0.09)	0.005
Agreeableness	−0.38 (0.12)	0.002
Extraversion	−0.16 (0.13)	0.199
Openness	−0.08 (0.13)	0.521
Psychosocial		
Anxiety (Beck)	0.48 (0.09)	<0.0001
Somatization (Whitely)	1.81 (0.40)	<0.0001
IBS-SSS	0.03 (0.01)	0.001
Demographics		
Age	0.05 (0.06)	0.452
Gender (F)	1.87 (1.95)	0.338

In weeks 1, 2, and 3 (*N* = 87, 82, 61, respectively) patients who completed the complaint survey were also genotyped. The distribution of patients in week 1 by COMT rs4680 val15met (χ^2^= 0.07, *P *=* *0.791) was in HWE with 21% of the patients having the met/met genotype, 51% val/met, and 28% val/val (Table 1). A significant main effect of COMT met alleles (β = −32.5, SE 14.1, *P *=* *0.0212) was qualified by COMT met alleles × conscientiousness (β = 0.73, SE 0.3, *P *=* *0.0042) and COMT met alleles × anxiety interactions (β = −0.42, SE 0.16, *P *=* *0.0078) (Table 4). The COMT met allele × neuroticism (β = 0.27, SE 0.27, *P *=* *0.3) and COMT met allele × agreeableness interactions (β = −0.008, SE 0.28, *P *=* *0.9784) were not significant. We did not correct for multiple testing, and as such the results are hypothesis-generating and require replication.

**Table 4:** Multivariable regression model of correlates of complaint ratings

Covariate	Beta (SE)	*P*-value
Main effects		
Conscientiousness	−0.87 (0.22)	0.0001
Anxiety	0.76 (0.13)	<0.0001
COMT met allele^1^	−32.5 (14.1)	0.0212
Interaction effects		
Conscientiousness × COMT met allele^2^	0.73 (0.26)	0.0042
Anxiety × COMT met allele^3^	−0.42 (0.16)	0.0078
Neuroticism × COMT met allele^4^	0.27 (0.27)	0.3125
Agreeableness × COMT met allele^5^	−0.008 (0.28)	0.9784

1 Reference COMT val/val.

2 Reference conscientiousness × val/val.

3 Reference anxiety × val/val.

4 Reference neuroticism × val/val.

5 Reference agreeableness × val/val.

## Discussion

The findings reported here potentially broaden our understanding of the factors that contribute to nonspecific clinical complaints by including the personality dimension of conscientiousness. As expected, we also found that neuroticism and baseline IBS symptom severity were associated with complaints. We additionally observed that the relationship between complaint severity reports and personality was modified by genetic variation in COMT.

Catechol-*O*-methyltransferase degrades catecholamines such as dopamine, epinephrine, and catechol estrogen resulting in its central role in cognitive (Mattay et al. 2003), sympathetic (Stein et al. 2005), cardiovascular (Hall et al. 2014), and endocrine networks (Kanasaki et al. 2008). This centrality accounts for the multiplicity of behavioral, clinical, personality, cognitive, and pain phenotypes influenced by genetic variation in COMT. While many studies report that COMT is associated with neuroticism (Stein et al. 2005), we did not find that COMT modified the association between neuroticism and complaint reporting. One explanation for this difference is that our subject pool consists of IBS patients with a Rome II IBS Symptom Severity Score of greater than 150 (Kaptchuk et al. 2008) and the relationship between COMT and neuroticism which has not been previously reported in this population could be considerably different than in a healthy population. Although personality and genotype are stable traits, the significant COMT association with conscientiousness and decreased complaint severity suggests a dynamic interaction between baseline personality traits and the active perception and articulation of a complaint. In this context COMT's ability to affect both dopamine and epinephrine regulation makes it a potentially pivotal target in rebalancing these systems to alleviate complaints.

Although this complaint survey gives equal weight to the presence and severity of each symptom, the significant association of this measure with the Whitely Index of somatization and the associations with anxiety and neuroticism are consistent with the literature and support the utility of the measure used here. This study is limited by its small size (*N* = 187 and 82 for the genetic subanalysis) and the homogeneity of the population studied. Since gender has known association with NEO personality traits, with women often scoring higher on conscientiousness (Costa et al. 2001) and since COMT allele frequencies vary by ethnicity, the results are not readily generalizable beyond complaint reporting in white female IBS patients, and the detection of a gene–personality interaction effect in such a small sample requires replication. Furthermore, as the NEO-FFI domains were originally defined by unit-weighting (Gorsuch 1983), they are highly correlated, and interactions of multiple traits with a gene may be a statistical artifact. Associations found between NEO categories and the other psychosocial characteristics measured in this study are also strong, particularly between neuroticism and anxiety (Jylha and Isometsa 2006) which contextualizes the multiple significant associations found here.

The issue of when a sensation becomes a complaint is integral to the question of what makes persons become patients (Eisenberg 1980). Further research on the contribution of individual personality and genetic composition is warranted to account for any subset of complaints attributable to background factors that are amplified and can undermine treatment.
